# Interaction and oxidative damage of DVDMS to BSA: a study on the mechanism of photodynamic therapy-induced cell death

**DOI:** 10.1038/srep43324

**Published:** 2017-03-02

**Authors:** Li Li, Huiyu Wang, Haiping Wang, Lijun Li, Pan Wang, Xiaobing Wang, Quanhong Liu

**Affiliations:** 1 Key Laboratory of Medicinal Resources and Natural Pharmaceutical Chemistry, Ministry of Education, National Engineering Laboratory for Resource Developing of Endangered Chinese Crude Drugs in Northwest of China, College of Life Sciences, Shaanxi Normal University, Xi’an 710119, Shaanxi, China; 2 Department of Ultrasound, Beijing Shijitan Hospital Affiliated to the Capital Medical University, 100038, Beijing, China; 3 Department of Surgical Oncology, Beijing Shijitan Hospital Affiliated to the Capital Medical University, 100038, Beijing, China

## Abstract

Photodynamic therapy (PDT) is a promising method for neoplastic and nonneoplastic diseases. In this study, we utilized sinoporphyrin sodium (DVDMS) as a sensitizer combined with light to investigate its cytotoxic effect on different cell lines. For this purpose, we chose bovine serum albumin (BSA) as a model to explore the mechanism of PDT-induced cell death at a molecular level. Our findings indicated that the combined treatment significantly suppressed cell survival. Fluorescence spectroscopy revealed a strong interaction between DVDMS and BSA molecules in aqueous solution, affecting DVDMS’ targeting distribution and metabolism. Spectroscopic analysis and carbonyl content detection indicated that DVDMS-PDT significantly enhanced the damage of BSA at a higher extent than Photofrin II-PDT under similar experimental conditions. Our observations were consistent with the cytotoxicity results. Excessive reactive oxygen species (ROS) were induced by the synergy effect of the sensitizer and light, which played an important role in damaging BSA and tumor cells. These results suggested that the interaction and oxidative damage of protein molecules by DVDMS were the main reasons to cell death and constitute a valuable reference for future DVDMS-PDT investigations.

Photodynamic therapy (PDT) has been increasingly applied in the anti-cancer field as a clinically approved and minimally invasive therapeutic procedure^1^,^2^,^3^. The technique is based on the specific light-activation of light-sensitive molecules called photosensitizers administered at non-toxic concentrations, and which preferentially accumulate in tumor cells. The reactive photosensitizer under an excited state, subjected to photon absorption, can transfer an electron to adjacent molecules. This mechanism, is referred to the type I photochemical reaction. Alternatively, a type II reaction involves excitation of molecular oxygen to the triplet state, transferring energy to the ground state molecular oxygen to produce reactive oxygen species (ROS), especially singlet oxygen (^1^O_2_)^4^,^5^,^6^. The subsequent ROS produced during the photochemical reaction ultimately lead to tumor cell death and tumor tissue destruction^7^.

Sensitizers are strategic components in PDT. They exhibit remarkable photophysical properties, including high stability, water-solubility, high phototoxicity, and low dark toxicity^8^,^9^,^10^. Porfimer sodium (Photofrin II) was the first photosensitizer approved by the US Food and Drug Agency and has already been applied clinically to treat various cancers and non-cancer diseases^11^,^12^,^13^,^14^. However, patients treated with Photofrin II suffer from a skin photosensitivity, requiring protection from sunlight or bright lights exposure for up to 3 months^15^. In addition, the complete mode of action is still not yet fully understood due to the unclear components of Photofrin II^11^. Therefore, novel onefold sensitizer is urgently needed for clinical PDT. Foscan and Tookad are novel photosensitizers, which show satisfactory antitumor effects as reported in clinical studies^16^,^17^. Nevertheless, Foscan can lead to perforation when treating hollow organs, although this risk can be lowered by reducing the dose. TOOKAD is not water soluble, so that it is administered in a Cremophor-based vehicle. Sinoporphyrin sodium (also called DVDMS) is the active ingredient of Photofrin II and gained independent intellectual property in China^12^,^18^,^19^. At a purity of above 98%, pharmacokinetic experiments showed that DVDMS preferentially accumulated in tumor tissues and metabolized quickly in normal tissues. Preliminary safety assessment indicated that DVDMS presented no obvious toxicity to major organs and had low skin-phototoxicity. Moreover, results from our previous work showed that DVDMS displayed higher activity than Photofrin II both *in vivo* and *in vitro* when appropriate ultrasound or light intensity was applied^18^,^20^,^21^. In addition, results of a meta-analysis indicated an increase of the mean survival time of 4T1 tumor-bearing mice after a DVDMS-PDT^15^. These data suggest that DVDMS represents a potential clinical photosensitizer, which deserves further explorations.

During a PDT process, generated ROS attack large biological molecules such as proteins, DNA, and lipid in cells^22^,^23^,^24^. Proteins are of paramount importance as they represent the ultimate controller and direct practitioners of life activities. They are involving in growth, development, metabolism, stress, energy conversion, and signal transduction of cells, among others^25^. When proteins are oxidized by ROS, their spatial structure is changed, leading to tragic changes in their biological functions, or even lost of activity. Ultimately, whole cells could die abnormally.

Bovine serum albumin (BSA) is often chosen as a model protein to investigate damages caused by drug interaction or other stimuli because of the low cost and easy accessibility^26^,^27^. Interaction reflects the degree and the way of combination between proteins and other molecules, and is conducive to reduce the distance and aggravate the degree of damage to protein by drug molecules^25^,^28^. In recent years, a large number of studies have shown the protein damaging effects of sensitizer in combination of light or ultrasound^28^,^29^,^30^,^31^. It could be predicted that, after combining with the appropriate sensitizer, light would play a crucial role in the damage of protein. It is therefore important to confirm the target biomolecules and select the appropriate sensitizers relevant for tumor therapy. Interestingly, the cellular responses to DVDMS-mediated PDT were mainly *in vitro* cell killing and *in vivo* tumor inhibition^15^,^32^,^33^. Unfortunately, the interaction and oxidative damages of DVDMS on protein molecules have not been studied yet. Further investigation is required to understand if the cytotoxicity induced by PDT is related to the oxidative damage by DVDMS of proteins.

Herein, we evaluated the cytotoxicity of DVDMS-mediated PDT in different cell lines and choose BSA as the model protein to fully explore the possible mechanisms of PDT-induced cell death at a molecular level. We hope the present study will contribute to the better understanding of the molecular mechanisms of DVDMS-PDT induced cell damage to improve the therapeutic efficiency of PDT.

## Results

### Cell viability assay

**Figure 1:**
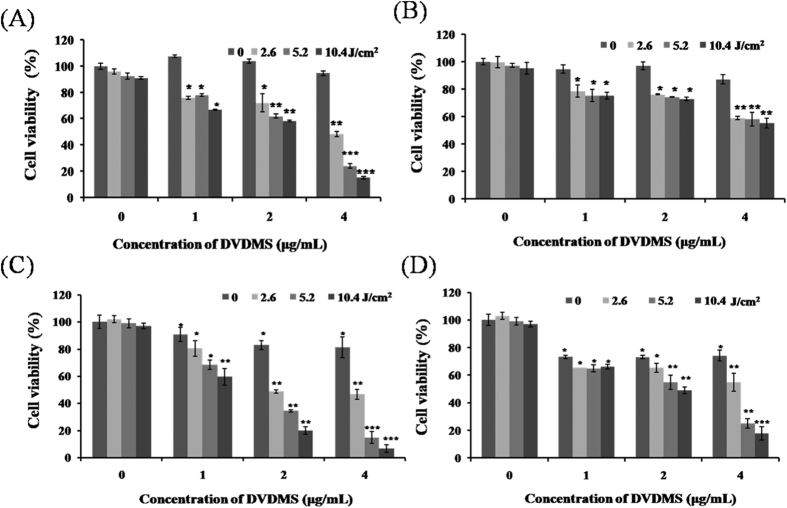
Cytotoxicity of DVDMS-mediated photodynamic therapy. Cell viability of DVDMS-PDT treatment on (**A**) SGC7901 cells, (**B**) Caco2 cells, (**C**) SW480 cells and (**D**) SW620 cells were measured by the MTT assay. Data are means ± SD of three independent experiments. *p < 0.05, **p < 0.01 and ***p < 0.001 versus untreated cells. MTT, 3-(4, 5-dimethylthiazol-2-yl)-2, 5-diphenyltetrazolium bromide.

Figure 1 shows the cell viability evaluated by 3-(4, 5-dimethylthiazol-2-yl)-2, 5-diphenyltetrazolium bromide tetrazolium (MTT) assay^10^ of DVDMS-PDT applied to different cell lines. Data in Fig. 1A revealed that the SGC7901 cells are viable. Both of groups, DVDMS alone (1, 2, and 4 μg/mL) and laser light alone (2.6–10.4 J/cm^2^), presented no inhibitory effect on cells. The cell viability decreased when the concentration of DVDMS and light dose increased. For example, when a light dose of 5.2 J/cm^2^ combined with 1, 2, and 4 μg/mL of DVDMS, the cell viability decreased down to 78.06%, 61.95%, and 24.00%, respectively. In the presence of 4 μg/mL of DVDMS, the cell viability losses were of 5.35% (p > 0.05), 51.57% (p < 0.05), 76.00% (p < 0.01), and 84.75% (p < 0.01) when the light dose were 0, 2.6, 5.2, and 10.4 J/cm^2^, respectively. The survival ratios of other cells such as Caco2 cells (Fig. 1B), SW480 cells (Fig. 1C), and SW620 cells (Fig. 1D) exhibited a similar behavior, just at different levels of the cell toxicity.

The Supplementary information of Fig. 1 shows the cell survival rate after Photofrin II-PDT. The results indicated that there was no inhibitory effect for both the Photofrin II only groups in the concentration range of 1–4 μg/mL and the PDT groups when the light doses were 0, 2.6, 5.2, and 10.4 J/cm^2^. To achieve similar PDT toxicity, much higher Photofrin II dosage was required (Supplementary Fig. 2), indicating that DVDMS was more effective than Photofrin II in cytotoxicity at the same weight ratio.

### Determination of intracellular ROS generation

**Figure 2:**
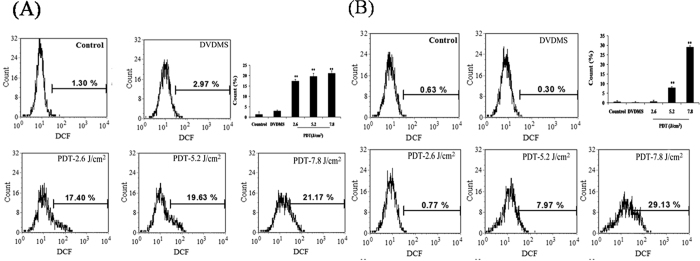
Determination of intracellular ROS generation in SW480 cells andSW620 cells after PDT treatment. (A) SW480 cells; (**B**) SW620 cells. The concentration of DVDMS was 2 μg/mL. Data shown are representative of three independent experiments. Results are presented as means ± SD of three independent experiments, SD is denoted by the error bars. *P < 0.05 represents PDT versus control.

The proposed mechanisms of PDT-induced cell death mainly focused on the generation of intracellular ROS. Therefore, we measured the intracellular ROS level using 2′, 7′- Dichlorodihydrofluo-rescein-diacetate (DCFH-DA) in SW480 cells and SW620 cells. The Fig. 2A shows the ROS level in SW480 cells treated with PDT. We observed 2.97%, 17.40% (p < 0.01), 19.63% (p < 0.01), and 21.17% (p < 0.01) of cells by measuring the DCF fluorescence of samples laser-light treated with 0, 2.6, 5.2, and 7.8 J/cm^2^, respectively. Moreover, the results presented in Fig. 2B revealed that the ROS levels in SW620 cells and DVDMS alone (2 μg/mL) showed no difference compared with the one of the control group (p > 0.05). In contrast, the ROS generation increased to 0.77% (p > 0.05), 7.97% (p < 0.01), and 29.13% (p < 0.01) for the PDT-treated cells when the light dose was 2.6, 5.2, and 7.8 J/cm^2^, respectively.

### Interaction between DVDMS and BSA

**Figure 3:**
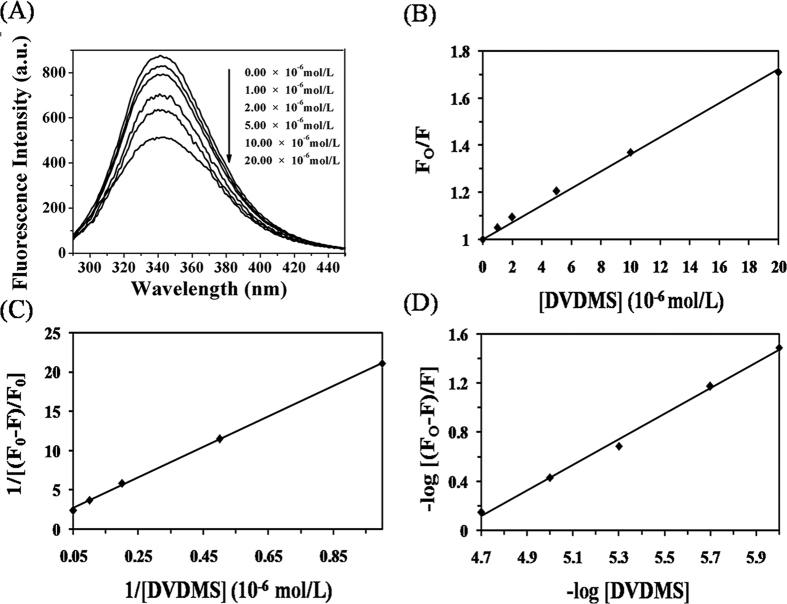
The binding parameters of DVDMS with BSA. Fluorescence spectra (**A**), Stern–Volmer plot (**B**), Lineweaver–Burk plot (**C**), and Double logarithm plot (**D**) of BSA solutions with different DVDMS concentrations (0.00–20.00 × 10^−6^ mol/L). ([BSA] = 2 mg/mL, pH = 7.40, T_solu_ = 37.00 ± 0.02 °C).

The interaction between the BSA molecules and small drugs could reduce the intrinsic fluorescence of BSA^34^. The data presented in Fig. 3A shows that the increase of the DVDMS concentration leaded to the gradual reduction of the fluorescence intensity of BSA, supporting the interaction between DVDMS and BSA molecules.

The following equations^34^,^35^,^36^ (Fig. 3B,C) are used to analyze the data of fluorescence intensity (Fig. 3A)


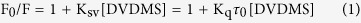



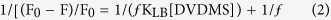



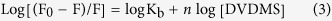



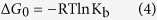


F_0_ and F represent the maximum fluorescence intensities in the absence and presence of DVDMS, respectively. K_SV_ is the dynamic quenching constant and [DVDMS] is the concentration of DVDMS. K_q_ is the bimolecular quenching rate constant and τ_0_ is the average lifetime of the fluorescence biomolecules in the absence of quencher and its value is about 10^−8^ s. ƒ is the fraction of accessible fluorescence and K_LB_ is the static fluorescence quenching association constant. K_b_ is the equilibrium constants and n is the binding site numbers. R and ΔG_0_ represent the gas constant (8.314 J mol^−1^ K^−1^) with the thermodynamic temperature T = 310 K (37.00 ± 0.02 °C) and the standard free energy, respectively. The corresponding results were listed in the Supplementary information Table 1.

The value of K_q_ obtained from our experiments was largely above 2 × 10^10^ L mol^−1^ s^−1^ 37, which indicated that static fluorescence quenching occurred between DVDMS and BSA. The linear regression expressed by Equation (2) better fitted the data (R^2^ = 0.999) than the Equation (1), resulting in a K_LB_ value of 8.812 × 10^4^ L mol^−1^ and a dissociation constant K_D_ of 1.135 × 10^−5^ calculated at 37 °C. The data confirmed that static fluorescence quenching occurred between the BSA and DVDMS molecules. Moreover, we could extract a K_b_ of 5.875 × 10^4^ L mol^−1^, indicating a strong interaction between the BSA and DVDMS. The determination of the number of binding site resulted in n = 1.040, suggesting that DVDMS and BSA interacted at least on one independent binding site. The negative sign of the ΔG_0_ value (−28.301 kJ mol^−1^) indicated that the binding of DVDMS to BSA molecule was spontaneous.

### The synchronous fluorescence spectra of BSA+ DVDMS solutions

**Figure 4:**
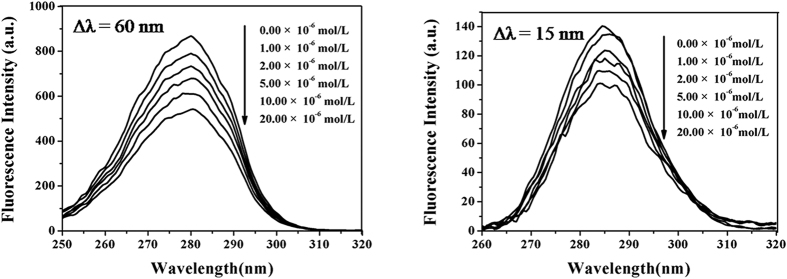
The synchronous fluorescence spectra of BSA + DVDMS solutions. Synchronous fluorescence spectra of BSA solutions with increasing of DVDMS concentrations (0.00 × 10^−6^ mol/L, 1.00 × 10^−6^ mol/L, 2.00 × 10^−6^ mol/L, 5.00 × 10^−6^ mol/L, 10.00 × 10^−6^ mol/L,20.00 × 10^−6^ mol/L). ([BSA] =2 mg/mL, pH = 7.40, T_solu_ = 37.00 ± 0.02 °C).

Synchronous fluorescence spectroscopy was applied to confirm characteristic information about Tyr residues or Trp residues^37^ and analyze the binding sites of DVDMS and BSA molecules. In addition, the Equation R_SFQ_ = 1 - F/F_0_ was used to calculate the decreasing percentages of synchronous fluorescence intensity. F_0_ and F were the synchronous fluorescence intensities of BSA solutions in the absence and presence of different DVDMS concentrations, respectively. The data summarized in Fig. 4 showed that the synchronous fluorescence intensities of BSA solutions for both Δλ = 15 and 60 nm gradually decreased as the DVDMS concentrations increased. But the R_SFQ_ (Supplementary Information Table 2) of data acquired at Δλ = 60 nm were more important than the corresponding ones at Δλ = 15 nm. These results suggested that the binding site between the DVDMS and BSA molecules was located closer to the Trp residues than the Tyr residues.

### UV–vis and fluorescence spectra of BSA solutions with DVDMS and Photofrin II

**Figure 5:**
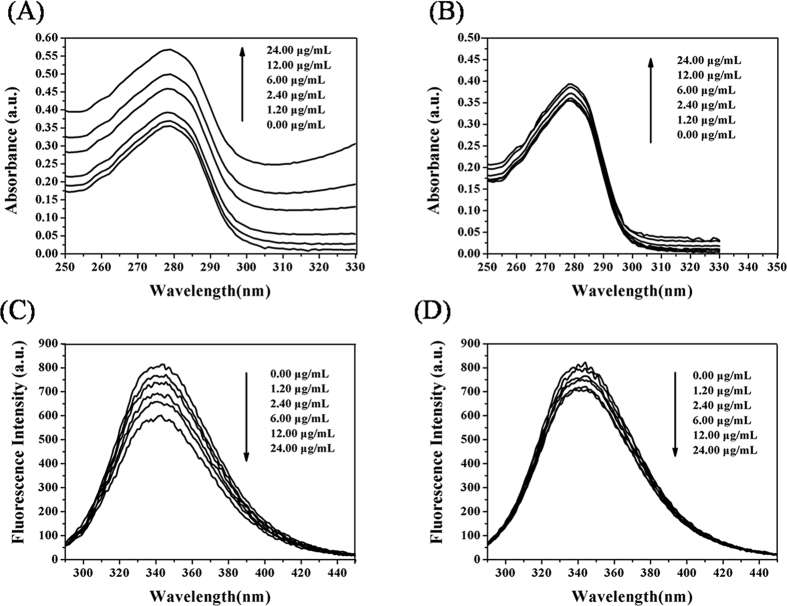
UV–vis and fluorescence spectra of BSA solutions with DVDMS or Photofrin II. UV–vis spectra (**A**) and fluorescence spectra (**C**) of BSA + DVDMS solutions, UV–vis spectra (**B**) and fluorescence spectra (**D**) of BSA + Photofrin II solutions. ([DVDMS] =[Photofrin II] =6 μg/mL, [BSA] = 2 mg/mL, pH = 7.40, T_solu_ = 37.00 ± 0.02 °C).

As a simple and applicable method, UV–vis absorption spectroscopy is often used to explore the interaction between drug molecules and BSA by monitoring the hyperchromic effect in case of structural damage of BSA^38^. As shown in the Fig. 5A, the absorption peak of BSA at 279 nm markedly increased as the DVDMS concentration increased. Data in Fig. 5B revealed that hyperchromicity was also found in the case of the Photofrin II-treated groups. However, we observed a less pronounced hyperchromicity of BSA in the case of the Photofrin II group compared with DVDMS-treated group. The intrinsic fluorescence intensity of the BSA solution decreased more obviously as the BSA molecules interacted with DVDMS in comparison with Photofrin II (Fig. 5C,D).

### UV – vis and fluorescence spectra of BSA under DVDMS-PDT and Photofrin II-PDT

**Figure 6:**
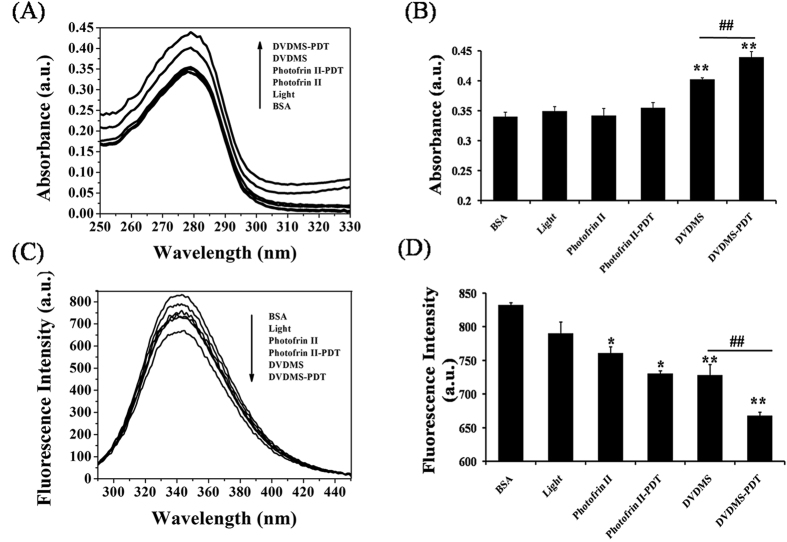
Changes of absorbance (**A** and **B**) and fluorescence intensity (**C** and **D**) of BSA solutions with different treatments. Light dose: 25 J/cm^2^, [DVDMS] = [Photofrin II] = 6 μg/mL, [BSA] = 2 mg/mL, pH = 7.40, T = 37.00 ± 0.02 °C, V_tota l_ = 0.4 mL. Error bars represent S.D. of the means from three independent experiments. *p < 0.05 and **p < 0.01 compared with BSA group, ^**##**^p < 0.01 between the groups.

As shown in the Fig. 6, a concentration of 6 μg/mL DVDMS or Photofrin II alone could not induce significant hyperchromicity and fluorescence quenching. Nonetheless, exposing BSA to laser light in the presence of 6 μg/mL of DVDMS or Photofrin II resulted in obvious hyperchromicity and fluorescence quenching, while no shift was observed in the protein fluorescence emission maximum. In addition, the hyperchromicity and fluorescence quenching of DVDMS-PDT samples were more obvious than the ones of Photofrin II–PDT samples.

### Protein Carbonyl Assay

**Figure 7:**
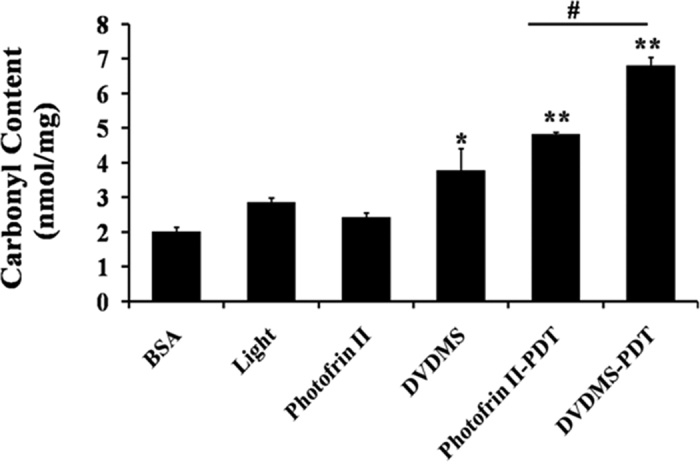
Carbonyl content in BSA with different treatments. Light dose: 50 J/cm^2^, [DVDMS] = [Photofrin II] = 6 μg/mL, [BSA] = 2 mg/mL, pH = 7.40, T = 37.00 ± 0.02 °C, V_total_ = 0.4 mL. Error bars represent S.D. of the means from three independent experiments.*p < 0.05 and **p < 0.01 compared with BSA group, ^**#**^p < 0.05 between groups.

The 2,4-Dinitrophenyl hydrazine (DNPH) method was used to evaluate the oxidative damage of protein by quantifying the quantity of protein carbonyls^39^,^40^,^41^. The total amounts of protein carbonyls in the different treatment groups are shown in the Fig. 7. The protein carbonyl content was 2.02 ± 0.003 nmol/mg in the control group. The addition of 12 μg/mL of DVDMS or Photofrin II increased the protein carbonyl content to 4.82 ± 0.003 nmol/mg (P < 0.05) and 2.43 ± 0.016 nmol/mg, respectively. When the laser light was applied, the protein carbonyl content increased to 6.80 ± 0.006 nmol/mg (P < 0.01) in the DVDMS-PDT group, which is much higher than that in the Photofrin II-PDT group (4.82 ± 0.002 nmol/mg (P < 0.01)).

### Detection of singlet oxygen generation

**Figure 8:**
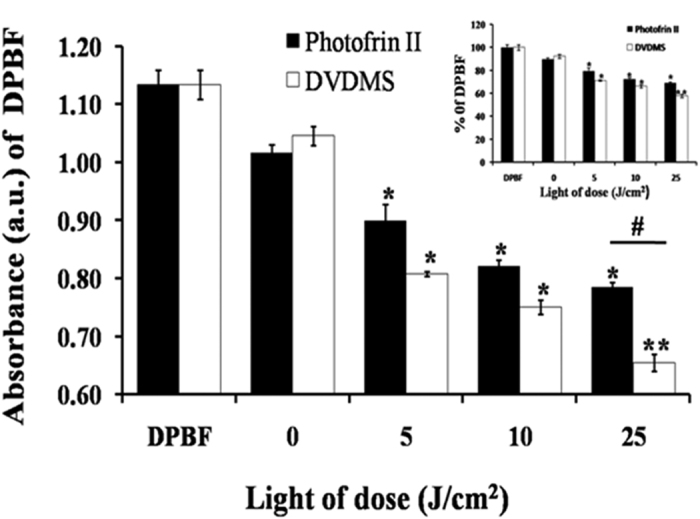
The detection of singlet oxygen generation. Absorbance of DPBF was analyzed by ultraviolet spectrophotometry after dissolution in 12 μg/mL DVDMS and Photofrin II in DPBF with light irradiation (0, 5, 10, 25 J/cm^2^). Error bars represent S.D. of the means from three independent experiments. *P < 0.05 and **P < 0.01 versus DPBF only group.

The yields of singlet oxygen production by DVDMS and Photofrin II were measured by the assay based on the photo-oxidation of 1, 3–diphenylisobenzofuran (DPBF) in anhydrous alcohol. As shown in Fig. 8, the addition of DVDMS and Photofrin II decreased the absorbance of DPBF compared with the one of the control by 7.76% (P > 0.05) and 10.32% (P > 0.05), respectively. After irradiated with different light dose (5, 10, and 25 J/cm^2^), the DPBF absorbance in the presence of 6 μg/mL of DVDMS, significantly decreased to 28.79% (P < 0.05), 33.86% (P < 0.05), and 42.26% (P < 0.01), respectively. When PDT was mediated by 6 μg/mL of Photofrin II, the absorbance of DPBF decreased gradually to 20.63% (P < 0.05), 27.60% (P < 0.05), and 30.78% (P < 0.05) when light dose were 5, 10, and 25 J/cm^2^, respectively. The singlet oxygen generation of Photofrin II was much lower than that of DVDMS under the same PDT dose treatment.

## Discussion

PDT is increasingly being recognized as a noninvasive therapeutic approach for a wide variety of cancers^3^,^42^,^43^,^44^. Currently, it is widely accepted that the choice of the sensitizers is one of the most essential factors in the success of PDT. The photosensitive molecule DVDMS, isolated from Photofrin II, is a newly developed sensitizer. Our previous studies provided the basic physico-chemical characteristics of DVDMS and we reported its *in vitro* and *in vivo* photo-activity^10^,^15^,^18^,^20^. The results indicated that DVDMS had a great clinical application prospect. Numerous preclinical and clinical studies have shown that PDT initiated a cascade of chemical reactions, involving ROS production, which triggered apoptosis, necrosis, and autophagy of cells^45^,^46^. Other studies reported its activation of the host immune system, which resulted in the generation of an acute inflammatory response^47^ and that it could hold back vascular damage for its possible recovery in the blood flow^48^. Recently, studies have explored the therapeutic effects and reaction mechanisms of PDT when utilizing tumor cells as assault target^2^,^8^,^13^,^15^,^32^,^33^. Wang Jun and others used spectroscopic methods to study the synergistic effect of the sensitizer with light or ultrasound on the macromolecular damaging of proteins and DNA and provided a basis for the molecular exploration of the photochemical activity of sensitizers^29^,^30^,^31^,^34^,^49^,^50^. Interaction and damaging of biological macromolecules became interesting approached in different cancer therapies^49^,^50^,^51^,^52^. The aim of this study was to investigate the cytotoxicity in different cancer cells and choose BSA as the model protein to explore the underlying molecular mechanism of DVDMS-PDT that induced cell death by protein damaging.

Our data indicated that DVDMS-triggered PDT applied on different tumor cells, presented distinct cytotoxicities depending on the utilized parameters, the cell types, and types of sensitizers. DVDMS could be effectively activated by light and displayed much higher phototoxicity than photofrin II (a clinically common used sensitizer) under the similar experimental conditions^9^,^10^. Proteins were expected to be the primary targets of ROS as to their abundance in cells (ca. 70% of the dry mass of most cells)^27^. An interaction or close proximity between the sensitizer and the primary targeting proteins during the photodynamic treatment was required since ROS could diffuse only in a perimeter of approximately 20 nm during its lifetime^25^. We showed that there was a strong interaction between BSA molecules and DVDMS. Moreover, the affinity between BSA and DVDMS was neither too strong nor too weak (K_b_ = 5.875 × 10^4^ L mol^−1^), which could lead to the DVDMS accumulation in tumor sites due to BSA interference with the pharmacokinetics and pharmacodynamics. The presented results perfectly corroborated our previous results from the metabolism study^15^,^53^.

Spectroscopy is an ideal tool to observe structural changes of proteins since it allows non-intrusive measurements of substances at low concentration under physiological conditions^30^. From evaluating hyperchromicity and fluorescence quenching, the application of laser light significantly improved the damaging extent of DVDMS on BSA and the damage level was much higher for DVDMS than for Photofrin II-PDT. The spectroscopic results were consistent with the cytotoxicity results. The interactions between DVDMS with BSA molecules could contribute to shorten the distance between the DVDMS and BSA molecules and prompt BSA to become more sensitive to ^1^O_2_, which could result in damaging hydrogen bonds, ionic bonds, hydrophobic interactions, –S-S– bonds, and aromatic ring stacking interactions, which maintained the secondary structures of the protein^54^. The damage of BSA could change the secondary structure (α-helix) and result in exposing the chromophoric amino acids such as tryptophan (Trp) and tyrosine (Tyr) to hyperchromicity and quenching of their intrinsic fluorescence^37^,^54^,^55^. In consequence, the physico-chemical properties of BSA could have been modified. Synchronous fluorescence spectrometry^52^,^53^ provided valuable information about the molecular microenvironment in the vicinity of the chromophore molecules and the drugs. The results shown in the Fig. 4 showed that the fluorescence intensities of both Tyr and Trp of BSA were affected, which indicated that the binding site of DVDMS to BSA molecules was closer to Trp residues than to Tyr residues. This observation supported indirectly that ROS damage sites were located near the Trp residues.

Numerous studies have demonstrated that PDT relies on the generation of ROS^12^,^13^,^14^,^15^. Our study also detected abundant intracellular and extracellular ROS generation after DVDMS-PDT treatment. The oxidative damage, improving the content of carbonyl protein, occurred in close proximity to the site where ROS were formed^56^,^57^,^58^. Since we have demonstrated that DVDMS presented a strong affinity to BSA, the generated ROS during the PDT could damage proteins and eliminate their biological function. The consequences could lead to biophysical, biochemical, and functional changes of the proteins and ultimately result to cell death.

Our study on the interaction between DVDMS and BSA contributed to the understanding of the *in vivo* DVDMS metabolism at the molecular level. The oxidative damage of the protein modified its structure that regulated cellular signal events, including apoptosis after PDT. We hoped that this study would support meaningful thought on tumor suppression and related molecular mechanism involved in PDT.

## Conclusion

In summary, this study evaluated the cytotoxicity induced by DVDMS-PDT on different cell lines. For this purpose, we chose BSA as the model protein to explore the possible mechanism of PDT-induced cell death at a molecular level. Our findings indicated that DVDMS spontaneously bound to BSA at a site much closer to Trp residues than Tyr residues. This interaction resulted in the static fluorescence quenching of BSA, supporting the strong interaction between BSA and DVDMS molecules. DVDMS-PDT exhibited superior BSA hyperchromicity, fluorescence quenching, and photoactivities, compared with photofrin II under similar experimental conditions in both cell free system or in intracellular activity detection. This work could be a valuable reference for further investigation in the synergy effect of DVDMS and light activation.

## Materials and Methods

All methods were performed in accordance with the approved guidelines.

### Chemicals

DVDMS was a gift from Fang from the Chinese Academy of Medical Sciences (Beijing, China). The molecular weight of DVDMS is 1230.6 with purity of 98.50%. It was dissolved in PBS (pH 7.2) with a storage concentration of 1 mM, and was stored in the dark at −20 °C.

MTT, DCFH-DA, DPBF, BSA, DNPH, Trichloroaceticacid (TCA) and Guanidine hydrochloride were purchased from Sigma Chemical Company (St Louis, MO, USA).All other reagents were commercial products of analytical grade.

### Cell culture

The human colorectal cancer SW-480, SW-620, Caco2 cells and human gastric cancer SGC7901 were obtained from the Cell Bank of the Chinese Academy of Science (Shanghai, China). SGC7901and SW480cells were cultured in Roswell Park Memorial Institute 1640 medium (SigmaAldrich, St Louis, MO, USA) containing 10% fetal bovine serum (Thermo Scientific Hyclone, Logan, UT, USA), 1% penicillin-streptomycin (penicillin 100 U/mL and streptomycin 100 g/mL), and 1% glutamine. Caco2 cells andSW620 cells were cultured in Dulbecco’s Modified Eagle’s Medium and L-15 medium (Gibco, Life Technologies, Carlsbad, CA, USA) containing 10% fetal bovine serum, 1% penicillin-streptomycin and 1% glutamine. All cells were maintained at 37 °C in humidified 5% CO^2^ atmosphere.

### PDT treatment protocols of cells

The semiconductor laser apparatus (excitation wavelength: 635 nm; manufacturer: Institute of Photonics & Photon Technology, Department of Physics, Northwest University, Shaanxi, China) was used in this study as the source of excitation light. Cells were seeded in 24-well culture plates (Corning Inc., NY, USA) and incubated at 37 °C with 5% CO_2_ to 80% of confluence. Then, all wells were randomly divided into 4 groups, namely control, DVDMS alone, light irradiation alone and PDT groups. For DVDMS alone and PDT groups, cells were incubated with various concentrations of DVDMS (1, 2, and 4 μg/mL) for 3 hours. Then, the cells were treated with different laser light dose ((2.6, 5.2, and 10.4 J/cm^2^) at a power intensity of 23.85 mW/cm^2^.

### Cell viability assay

Cell viability was analyzed in the different types of cells using conventional MTT assay^10^. Cell viability using the following equation:


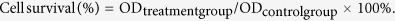


### Determination of intracellular ROS generation

DCFH-DA assay was applied to detect intracellular ROS^10^. A specific volume of 4 μM DCFH-DA was added to the cells of the different groups at 37 °C for 30 min before PDT. At 2 h after treatment, the cells of each group were harvested and then immediately analyzed by flow cytometry.

### Measurement of binding parameters

A solution of BSA (2 mg/mL) was prepared in highly pure deionized water (pH = 7.4) and stored at 4 °C. The BSA solutions were prepared by increasing DVDMS concentrations (0.00–20.00 × 10^−6^ mol/L) and used to monitor the intrinsic protein fluorescence (λ_ex_ = 278 nm and λ_em_ = 343 nm) using a LS55 fluorescence spectrophotometer (Perkin Elmer, USA). All test solutions were incubated for 30 min at 37.0 ± 0.02 °C before measurement. The maximum fluorescence intensity was used to calculate the binding parameters of BSA with DVDMS such as the dynamic quenching constant (K_SV_), the quenching rate constant of bimolecular (K_q_), the equilibrium constant (K_b_), and the binding site numbers (n)^52^.

### Analysis of synchronous fluorescence spectra

Synchronous fluorescence spectrometry^50^ was applied to confirm the binding sites of BSA and DVDMS. The synchronous fluorescence spectra of BSA solutions with different DVDMS concentrations were analyzed at Δλ = 15 nm for the Tyr residues and Δλ = 60 nm for the Trp residues. The corresponding ratios of synchronous fluorescence quenching (R_SFQ_ (%)) were calculated.

### UV – vis and fluorescence spectra of BSA under DVDMS-PDT and Photofrin II-PDT

The absorption spectra (UV–vis spectrophotometer, Beijing Purkinje General Instrument Co. Ltd, China) and fluorescence spectra (LS55 spectrofluorimeter, Perkin Elmer, USA) were determined in order to evaluate the damage to BSA. The BSA solution was divided randomly into six groups: (1) BSA, (2) BSA+DVDMS, (3) BSA+Photofrin II, (4) BSA+laser light, (5) BSA+DVDMS+ laser light, and (6) BSA+ Photofrin II+ laser light. The photosensitizer concentration was 6 μg/mL. Then, the BSA solutions were exposed to visible light (laser light dose: 25 J/cm^2^, power intensity: 53.79 mW/cm^2^).

### Protein Carbonyl Assay

The change of protein carbonyl content was used as the indicator of protein oxidative damage^29^,^59^. We monitored the absorption of 2, 4–dinitrobenzene hydrazone at 370 nm, which reflected the index of total protein carbonyl content. In this work, a volume of 0.4 mL of experimental sample in which the photosensitizer concentration was 12 μg/mL, was irradiated for 930 s, then mixed with 0.5 mL of ice-cold 20% TCA and centrifuged at 4 °C for 10 min. The protein precipitation was dissolved in 1 mL of a DNPH solution (10 mM in 2 M HCl) and incubated at 37 °C in the dark for 1 h with vortexing every 10 min. After the reaction, 0.5 mL of the 20% TCA solution was added to the reaction mixture, followed by centrifugation. The formed precipitate was washed three times with 1 mL of a mixture of ethyl acetate/ethanol (1:1, v/v) solution. Finally, the precipitate was re-suspended in 1 mL of a guanidine hydrochloride (6 M) solution and then centrifuged. The absorbance of the supernatant was then recorded at 370 nm.

### Detection of singlet oxygen generation

The yield of the production of singlet oxygen was measured by the photo-oxidation of DPBF dissolved in anhydrous alcohol. The reaction between DPBF and singlet oxygen triggers the oxidation of DPBF, leading to its corresponding diketone. As a result, DPBF was consumed and the absorbance of DPBF at about 410 nm reduced^53^. DVDMS or Photofrin II (6 μg/mL) dissolved in 0.2 mM DPBF was irradiated with different laser light (excitation wavelength 635 nm, laser light dose: 5, 10, 25 J/cm^2^, power intensity: 53.79 mW/cm^2^). The absorbance of DPBF at 414 nm was monitored using UV–vis spectrophotometer.

### Statistical analysis

Data are expressed as mean ± standard deviation of at least three independent experiments. Statistical analysis was performed by one-way analysis of variance. Statistical significance was established at a value of p < 0.05^10^,^15^,^18^.

## Additional Information

**How to cite this article:** Li, L. *et al*. Interaction and oxidative damage of DVDMS to BSA: a study on the mechanism of photodynamic therapy-induced cell death. *Sci. Rep.* **7**, 43324; doi: 10.1038/srep43324 (2017).

**Publisher's note:** Springer Nature remains neutral with regard to jurisdictional claims in published maps and institutional affiliations.

## Supplementary Material

Supplementary Information

Supplementary Table 1

Supplementary Table 2
