# Supplementary material for: Interaction and oxidative damage of DVDMS to BSA: a study on the mechanism of photodynamic therapy-induced cell death
Source: Sci Rep. 2017 Mar 2;7:43324. doi: 10.1038/srep43324 (PMC5333107; doi:10.1038/srep43324)

Supplementary Figures

Title: Interaction and oxidative damage of DVDMS to BSA: a study on the mechanism of photodynamic therapy-induced cell death

Authors: Li Li[1,#]

Huiyu Wang[2,#]

Haiping Wang[1, #]

LijunLi[3]

Pan Wang[1]

Xiaobing Wang[1,*]

Quanhong Liu[1,*]

Co - first authors.

*The corresponding author.

Figure 1. Cytotoxicity of Photofrin II-mediated photodynamic therapy. Cell viability of Photofrin II-PDT treatment on (A) SGC7901 cells, (B) Caco2 cells, (C) SW480 cells and (D) SW620 cells were measured by the MTT assay. Data are means ± SD of three independent experiments.

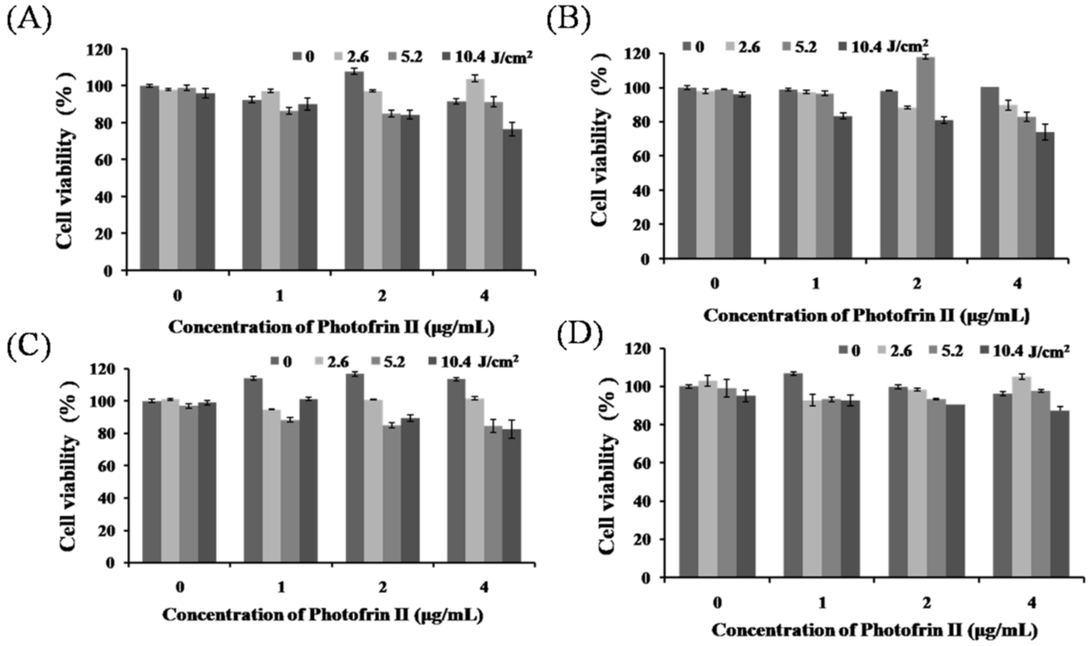

Figure 2. Cytotoxicity of Photofrin II-mediated photodynamic therapy. Cell viability of Photofrin II-PDT treatment on (A) SW620 cells, (B) SGC7901 cells, (C) Caco2 cells and were measured by the MTT assay. Data are means ± SD of three independent experiments.

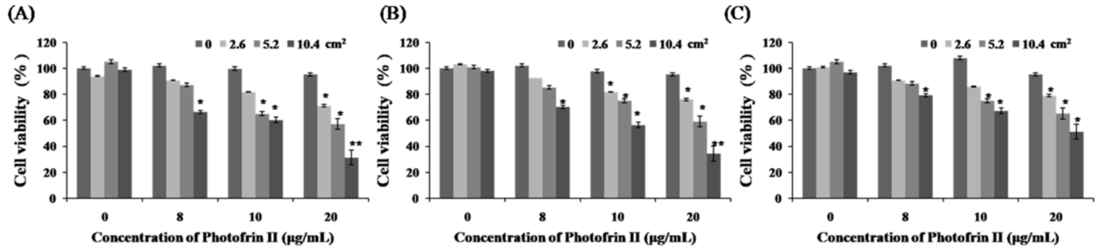

Supplement: Supplementary Information [file srep43324-s1.pdf]
