# Supplementary material for: Interaction and oxidative damage of DVDMS to BSA: a study on the mechanism of photodynamic therapy-induced cell death
Source: Sci Rep. 2017 Mar 2;7:43324. doi: 10.1038/srep43324 (PMC5333107; doi:10.1038/srep43324)
Supplement: Supplementary Table 1 [file srep43324-s2.pdf]

Supplementary information (Table 1**)**

Title: Interaction and oxidative damage of DVDMS to BSA: a study on the mechanism of photodynamic therapy-induced cell death

Authors: Li Li[1,#]

Huiyu Wang[2,#]

Haiping Wang[1, #]

LijunLi[3]

Pan Wang[1]

Xiaobing Wang[1,*]

Quanhong Liu[1,*]

Co - first authors.

*The corresponding author.

Table 1 Quenching constants (K_{SV} and K_q), the static fluorescence quenching association constant (K_{LB}), dissociation constants (K_D), the equilibrium constants (K_b) and binding site numbers (n) are calculated according to Stern–Volmer plots, Lineweaver–Burk plots and Double logarithm plots of BSA (2 mg/mL) + DVDMS (0.00–20×10^{-6} mol/L) , pH = 7.40, T_{solu}= 37.00 ±0.02 ℃

Stern – Volmer plot	R^2	K_{SV} (L mol^{-1})	Kq (L mol^{-1} s^{-1})	
F_0/F = 0.036 [DVDMS] + 1	0.995	3.600 ×10^4	3.600 ×10^{12}	
Lineweaver – Burk plot	R^2	*f*	K_{LB} (L mol^{-1})	K_D (L mol^{-1})
$1/[(F_0 – F)/F_0]$ = 19.450/[DVDMS] + 1.714	0.999	0.583	8.812 ×10^4	1.135 ×10^4
Double logarithm plot	R^2	K_b (L mol^{-1})	n	ΔG_0 (kJ mol^{-1})
$\log[(F_0 – F)/F]$ = 1.040 log[DVDMS] + 4.769	0.996	5.875 ×10^4	1.040	-28.301
