# Supplementary material for: Interaction and oxidative damage of DVDMS to BSA: a study on the mechanism of photodynamic therapy-induced cell death
Source: Sci Rep. 2017 Mar 2;7:43324. doi: 10.1038/srep43324 (PMC5333107; doi:10.1038/srep43324)
Supplement: Supplementary Table 2 [file srep43324-s3.pdf]

Supplementary information (Table 2)

Title: Interaction and oxidative damage of DVDMS to BSA: a study on the mechanism of photodynamic therapy-induced cell death

Authors: Li Li[1,#]

Huiyu Wang[2,#]

Haiping Wang[1, #]

LijunLi[3]

Pan Wang[1]

Xiaobing Wang[1,*]

Quanhong Liu[1,*]

Co - first authors.

*The corresponding author.

Table 2 Synchronous fluorescence quenching ratio (R_{SFQ}) of BSA + DVDMS (DVDMS : 0.00×10^{-6} mol/L, 1.00×10^{-6} mol/L, 2.00×10^{-6} mol/L, 5.00×10^{-6} mol/L, 10.00×10^{-6} mol/L, 20.00×10^{-6} mol/L) ([BSA] = 2 mg/mL, pH = 7.40, T_{solu} = 37.00 ± 0.02 ℃).

DVDMS concentration/(10^{-6} mol/L)	R_{SFQ} (%)	
	Δλ=60nm	Δλ=15nm
0	0	0
1	8.964	1.613
2	15.520	9.315
5	21.810	16.014
10	29.702	22.690
20	39.340	29.754
